# Diagnosis of periprosthesis joint infection and selection of replantation timing: a novel nomogram diagnosis model

**DOI:** 10.1097/JS9.0000000000002067

**Published:** 2024-09-05

**Authors:** Jianye Yang, Leilei Qin, Chen Zhao, Hai Wang, Cheng Chen, Tao Zhang, Bo Zhu, Li Wei, Xudong Su, Yujian Li, Ning Hu, Wei Huang

**Affiliations:** a Department of Orthopedics, The First Affiliated Hospital of Chongqing Medical University; b Orthopedic Laboratory of Chongqing Medical University; c Department of Orthopedics, Chongqing University Fuling Hospital, Chongqing, People’s Republic of China

**Keywords:** diagnosis model, inflammatory marker, nomogram, prosthetic joint infection, synovial fluid

## Abstract

Preoperative diagnosis of periprosthetic joint infection (PJI) is critical to guide treatment options and improve patient outcomes. In this letter, the authors discuss results from our experiences with a novel nomogram diagnosis model based on serum and synovial fluid indicators for the preoperative diagnosis of PJI. The results showed that the novel nomogram diagnosis model can distinguish PJI from aseptic loosening before the operation. And it is also a useful candidate for the selection of the timing of current secondary revision.

Highlights

- This is the first study to establish and validate a nomogram diagnostic model based on serum and synovial fluid indicators for clinicians to distinguish between PJI and aseptic loosening.
- In this work, we determined that serum indicators, including D-Dimer, serum interleukin-6 (IL-6), and synovial fluid indicators including IL-1β, IL-4, IL-6, CD64 index, PMN%, C-reactive protein were the main factors associated with PJI in patients.
- The developed nomogram diagnostic model is also a useful candidate for the selection of the timing of the current secondary revision.

A periprosthetic joint infection (PJI) can be a devastating complication of total joint arthroplasty, which is becoming more and more common and often resulting in a long treatment process^1^. The differential diagnosis between PJI and aseptic prosthetic loosening is crucial, as it determines the correct subsequent surgical strategy for the patient^2^. However, there is still a lack of a single diagnostic test that can accurately diagnose or rule out PJI^3^.

In recent years, a synovial fluid (SF) biomarker has become increasingly popular due to its low cost, ease of interpretation, and accuracy^3,4^. In addition, due to the nomogram’s higher accuracy and easier comprehension, this has led to the increasing use of it by patients and physicians alike to aid clinical decision-making^5^. Therefore, we collected serum (SE) and synovial fluid indicators from revision patients to develop a novel nomogram diagnosis model based on these indicators that is easier to operate and generalize. We hope that this diagnostic strategy will help clinicians and patients diagnose or rule out infection before the operation and choose the appropriate time for the second-stage revision.

The research is part of our registered project on the Chinese Clinical Trial Registry (ID: ChiCTR1800020440). The study was undertaken according to the STROBE guidelines^6^ and the STARD criteria^7^. From January 2015 to December 2021, we retrospectively collected the patients who were admitted for joint revision and randomly divided them into a 7:3 training and validation cohorts. LASSO regression analysis and univariate and multivariate logistic regression analysis were performed to select the most suitable diagnostic index. A nomogram model was built based on filtered indicators. And assessing the diagnostic performance and clinical usability of the nomogram using receiver operating characteristic (ROC) curve, calibration curves (CAC), decision curve analysis (DCA), and clinical impact analysis (CIA). In addition, we prospectively collected revision cases to externally validate the nomogram diagnostic model.

Continuous variables with normal distributions were presented as means and SD. Otherwise, the variables are described using interquartile ranges. We choose appropriate statistical methods based on the characteristics of the data.

We analyzed 422 cases (training cohort: 295, validation cohort:127), and 128 cases (30.33%) were diagnosed with PJI. The demographic characteristics and clinical data of the training and validation cohort are shown in Supplemental Tables 1 and 2 (Supplemental Digital Content 1, http://links.lww.com/JS9/D394). In the training cohort, LASSO regression analysis was used to screen out D Dimer (DD), SE-IL-6, SE-CRP, ESR, CD64 index, SF-CRP, IFN-γ, SF-IL-1β, SF-IL-4, SF-IL-6, SF-IL-8, SF-IL-12, and PMN% as potential diagnostic indicators (Supplemental Figure 1, Supplemental Digital Content 1, http://links.lww.com/JS9/D394). The above 14 variables were subjected to univariate and multivariate logistic regression analysis, and we found that SE-IL-6, DD, CD64, SF-CRP, SF-IL-1β, SF-IL-4, SF-IL-6, and PMN% may have important significance in diagnosing the diagnosis of PJI (Supplemental Table 3, Supplemental Digital Content 1, http://links.lww.com/JS9/D394).

Based on the above results, we further constructed the diagnostic nomogram by combining the interaction among SE-IL-6, DD, CD64, SF-CRP, SF-IL-1β, SF-IL-4, SF-IL-6, and PMN% (Fig. 1). In Figure 2A, CAC of PJI probabilities (C-index: 0.998, Hosmer–Lemeshow: *χ* ^2^=6.3909, df=8, *P*=0.604) show good agreement between nomogram predictions and actual observations in the training cohorts. The result of DCA indicated that using this nomogram to predict the probability of the diagnosis of PJI would yield a greater net benefit than an all-or-no patient intervention scenario if the threshold probability is in the range of 0.01–1 (training cohort) and 0.02 to 1 (validation cohort), suggesting a high value for clinical application (Fig. 2B). Next, we used CIA to evaluate the stratification of the Nomogram’s correct probability of diagnosing 1000 patients (Fig. 2C). When the threshold probability is greater than 0.2, the predicted number of PJI patients is very close to the actual number of PJI patients, and the cost-to-benefit ratio at this time is 0.25. AUC values for the training cohort and validation cohort are 0.998 (95% CI: 0.995–1, sensitivity:0.978, specificity:0.990) and 0.997 (95% CI: 0.993–1, sensitivity:0.971, specificity:0.967), respectively, suggesting that the nomogram has good diagnostic performance (Fig. 2D). Furthermore, we found that the diagnostic performance of the nomogram was significantly higher than that of a single diagnostic indicator (Fig. 2E, F). The basic characteristics of the external validation cohort are shown in Supplemental Table 4 (Supplemental Digital Content 1, http://links.lww.com/JS9/D394). In the PJI cohort, diagnosis using the nomogram diagnosis model found that the true positive rate is 96.491% (Table 1). Overall, the nomogram diagnosis model has higher accuracy and sensitivity relative to MSIS (2013) and ICM (2018) when performing preoperative diagnosis and has similar specificity.

**Figure 1:**
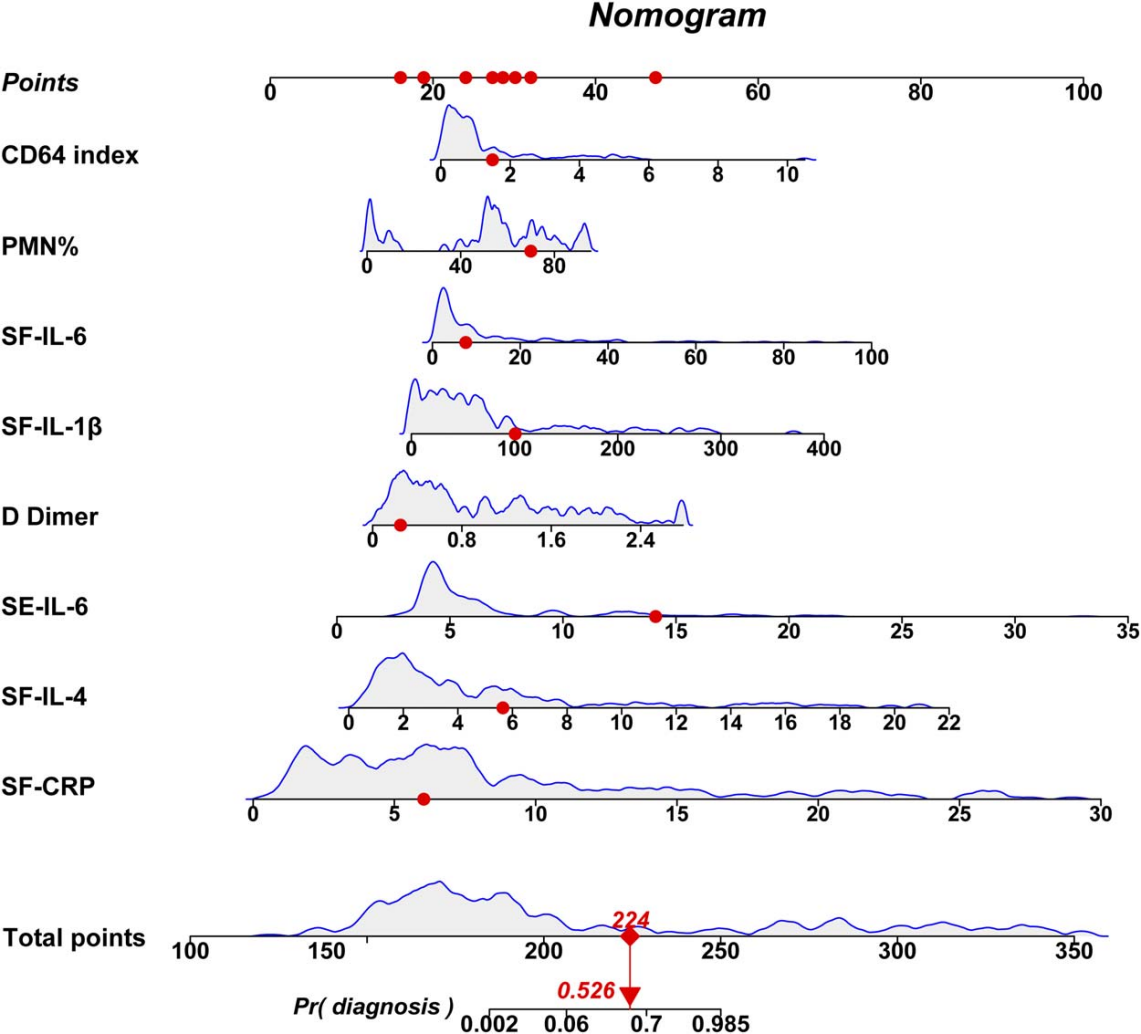
Nomogram predicting the confirmed diagnosis of PJI probability in the joint revision patients. Gray density plots describe the distribution of the joint revision patients in diagnosis parameters and total points while the red dots represent one patient’s points as an example.

**Figure 2:**
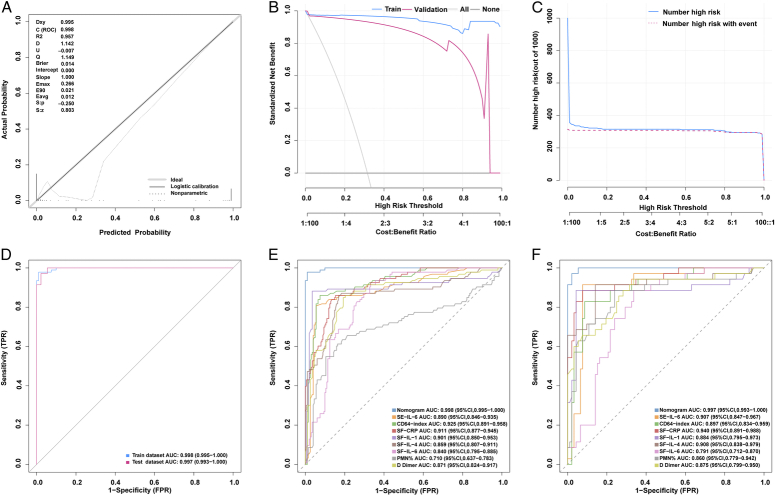
Evaluating nomogram diagnosis model performance. (A) Calibration curves for the predicting probability of diagnosed with PJI in the training cohort, all *P*-value >0.05 in the Hosmer–Lemeshow test suggested an agreement between the predicted probabilities and observed outcome. (B) The decision curve compares the net clinical benefits of three scenarios in predicting the confirmed diagnosis of PJI probability: a perfect prediction model (grey line), screen none (horizontal solid black line), and screen based on the nomogram (blue and red line). (C) Clinical impact curve to predict the confirmed diagnosis of PJI for a population size of 1000 based on Train cohort. (D)The ROC for performance to distinguish individuals with PJI from aseptic in the training cohort and validation cohort. Diagnostic performance comparison between Nomogram and single indicator in the training cohort (E) and validation cohort (F).

**Table 1:** Performance of the nomogram diagnosis model with the traditionally used musculoskeletal infection society (MSIS) and international consensus meeting (ICM) criteria before the surgery.

Variable	Train					Test			
	Aseptic (*N*=202)		PJI (*N*=93)		*P*	Aseptic (*N*=92)		PJI (*N*=35)	*P*
Criteria	PJI cohort (*N*=57)		Aseptic cohort (*N*=89)		Accuracy	Sensitivity	Specificity	Positive predictive value	Negative predictive value
	True positives	False negatives	True negatives	False positives					
MSIS (2013)	40	17	88	1	87.671%	70.175%	98.876%	97.561%	83.810%
ICM (2018)	49	8	88	1	93.836%	85.965%	98.876%	98.000%	91.667%
Nomogram	55	2	88	1	97.945%	96.491%	98.876%	98.214%	97.778%

Although, MSIS (2013) and ICM (2018) have made great contributions in establishing standardized and unified diagnostic criteria for PJI. But PJI lacks the gold standard for absolute accuracy, especially before revision joint surgery. In this case, the diagnosis of PJI needs to rely on a combination of multiple diagnostic indicators^8^. The nomogram diagnostic model we constructed is a combination of serology and synovial fluid indicators, which has higher sensitivity and specificity than traditional serology or synovial fluid diagnostic methods. And this combination comprehensively considered the different weights carried the interactions between the results of all diagnostic tests in diagnosing PJI. It must be mentioned that this interaction affects the performance of the diagnostic test in a significant way^9^. The nomogram model we constructed allows clinicians to draw well-founded conclusions before undertaking more expensive and invasive. Furthermore, it screens out the most important diagnostic indicators, minimizing unnecessary testing of low sensitivity and specificity and potentially reducing the cost. In summary, our scoring system can complete preoperative diagnosis more accurately and has obvious advantages, even in some cases that are difficult to exclude by traditional methods. In the future, by continuously including PJI cases from multiple centers, the diagnostic performance of the nomogram will be further improved.

In summary, our nomogram for diagnosing PJI showed good discrimination based on multiple diagnostic indicators, force, and calibration capabilities. The application of the nomogram will help clinicians to better diagnose PJI and make timely and reasonable decisions to optimize the treatment plan and benefit the patients. However, there are still large-scale prospective studies that need to be conducted before this quantitative tool can be greatly generalizable.

## Ethical approval

This study was approved by the Ethics Committee of the First Affiliated Hospital of Chongqing Medical University (No.20187101).

## Consent

Written informed consent was not relevant to this manuscript.

## Source of funding

This research was financially supported by the National Natural Science Foundation of China (82372425 and 82072443), the National Natural Science Foundation of Chongqing, China (CSTB2023NSCQ-MSX0166), Postdoctoral Fellowship Program of CPSF (GZC20233351), and Chongqing postdoctoral research project special support (2023CQBSHTB3124).

## Author contribution

J.Y., L.Q.: conceptualization, data curation, formal analysis, methodology, software, writing – original draft, and writing – review and editing; C.Z.: conceptualization, data curation, formal analysis, investigation, methodology, resources, and visualization; H.W.: conceptualization, data curation, formal analysis, investigation, methodology, validation, and visualization; C.C.: conceptualization, data curation, formal analysis, methodology, and resources; T.Z.: conceptualization, investigation, resources, software, supervision, and validation; B.Z.: data curation, formal analysis, investigation, and methodology; L.W.: data curation, investigation, methodology, resources, and software; X.S.: conceptualization, data curation, formal analysis, investigation, and methodology; N.H.: conceptualization, formal analysis, project administration, resources, supervision, validation, and writing – review and editing; W.H.: conceptualization, funding acquisition, project administration, supervision, validation, visualization, writing – review and editing.

## Conflicts of interest disclosure

The authors declare no conflicts of interest.

## Research registration unique identifying number (UIN)

1. Name of the registry: Chinese Clinical Trial Registry.
2. Unique identifying number or registration ID: ChiCTR1800020440.
3. Hyperlink to your specific registration (must be publicly accessible and will be checked): https://www.chictr.org.cn/.

## Guarantor

Ning Hu and Wei Huang accept full responsibility for the work and/or the conduct of the study, had access to the data, and controlled the decision to publish. E-mail: huncqjoint@yeah.net; huangwei68@263.net.

## Data availability statement

The authors confirm that the data supporting the findings of this study are available within the article and its supplementary materials. Also, any extra data of this study are available on request from the corresponding author.

## Provenance and peer review

Not commissioned, externally peer reviewed.

## Supplementary Material
