# Supplementary material for: Diagnosis of periprosthesis joint infection and selection of replantation timing: a novel nomogram diagnosis model
Source: Int J Surg. 2024 Sep 5;111(1):1591–5. doi: 10.1097/JS9.0000000000002067 (PMC11745575; doi:10.1097/JS9.0000000000002067)
Supplement: Supplementary file 1 [file js9-111-1591-s001.docx]

**Supplemental Table 1. Comparison of demographics and clinical characteristics between the training and validation cohort.**

| **Variable** | | **Overall (N=422)** | **Training cohort (N=295)** | **Validation cohort (N=127)** | **P** |
| --- | --- | --- | --- | --- | --- |
| **Joint (%)** | **Hip** | 215 (50.948%) | 43 (46.237%) | 0.602 | 0.125 |
|  | **Knee** | 207 (49.052%) | 72 (56.693%) |  |  |
| **sex (%)** | **Male** | 190 (45.024%) | 55 (43.307%) | 0.804 | 0.541 |
|  | **Female** | 232 (54.976%) | 60 (47.244%) |  |  |
| **Age (years)** | | 66.000(59.000, 73.750) | 66.000(59.000,74.000) | 67 (52.756%) | 0.356 |
| **Weight (Kg)** | | 60.000(55.000, 70.000) | 60.000(54.625,69.750) | 66.000(59.000,73.000) | 0.517 |
| **BMI (Kg/m2)** | | 23.685(21.100, 25.955) | 23.650(20.883,25.810) | 60.000(54.000,69.000) | 0.523 |
| **WBC (*109/L)** | | 5.885(4.942, 7.300) | 5.888(4.890,7.398) | 23.880(20.240,25.510) | 0.005 |
| **HB (g/L)** | | 121.000(108.000, 133.000) | 120.000(106.000,132.000) | 5.710(4.773,7.006) | 0.712 |
| **PLT (*109/L)** | | 143.000(120.000, 219.500) | 179.465(125.032,246.000) | 119.280(106.000,129.995) | 0.017 |
| **ANG (*109/L)** | | 3.690(2.715, 4.620) | 3.730(2.782,4.797) | 201.170(130.000,255.485) | 0.003 |
| **AL(*10^9^/L)** | | 1.670(1.270, 2.315) | 1.740(1.280,2.498) | 3.690(2.800,4.570) | 0.392 |
| **N% (%)** | | 59.500(54.025, 67.125) | 61.750(55.578,69.095) | 1.820(1.330,2.595) | <0.001 |
| **L %(%)** | | 25.950(18.700, 31.250) | 25.715(18.300,30.375) | 61.900(55.725,69.140) | 0.041 |
| **DD (mg/ml)** | | 0.530(0.300, 0.970) | 0.700(0.350,1.440) | 26.000(17.860,29.235) | <0.001 |
| **SE-IL-6 (ng/ml)** | | 4.370(4.060, 5.150) | 4.820(4.170,6.580) | 0.570(0.280,1.180) | <0.001 |
| **SE-CRP (mg/ml)** | | 4.490(3.500, 6.415) | 5.935(3.885,18.925) | 4.780(4.140,7.290) | <0.001 |
| **ESR (mm/h)** | | 27.500(15.000, 52.000) | 33.000(16.000,62.855) | 5.760(3.750,12.325) | <0.001 |
| **CD64** | | 0.490(0.233, 0.808) | 0.710(0.320,1.445) | 32.000(15.000,63.500) | <0.001 |
| **SF-CRP (mg/ml)** | | 5.035(2.887, 6.982) | 6.280(3.677,10.305) | 0.630(0.299,1.195) | <0.001 |
| **IFN-γ (pg/ml)** | | 4.115(1.487, 16.665) | 4.695(1.652,18.730) | 5.700(3.550,10.300) | 0.023 |
| **SF-IL-1β (pg/ml)** | | 32.670(15.262, 53.340) | 45.743(18.832,90.458) | 4.730(2.005,19.065) | <0.001 |
| **SF-IL-2 (pg/ml)** | | 7.800(3.018, 13.568) | 6.945(2.768,13.605) | 44.538(15.249,81.940) | 0.415 |
| **SF-IL-4 (pg/ml)** | | 2.350(1.603, 3.738) | 3.048(1.750,6.040) | 6.090(2.325,13.890) | <0.001 |
| **SF-IL-6 (ng/ml)** | | 3.175(2.050, 6.917) | 5.065(2.450,12.357) | 2.876(1.562,5.945) | <0.001 |
| **SFIL-8 (pg/ml)** | | 786.280(160.850, 1378.445) | 651.270(126.900,1324.572) | 5.070(2.726,10.945) | 0.899 |
| **SF-IL-10 (pg/ml)** | | 2.285(1.255, 3.997) | 2.170(1.183,4.482) | 628.610(125.095,1278.265) | 0.455 |
| **SF-IL-12 (pg/ml)** | | 1.765(0.940, 4.103) | 1.685(0.820,3.895) | 2.100(0.985,4.415) | 0.305 |
| **SF-IL-17 (pg/ml)** | | 1.500(0.780, 4.415) | 1.500(0.742,7.835) | 1.610(0.915,3.265) | <0.001 |
| **PMN% (%)** | | 52.420(32.957, 59.580) | 55.330(38.532,71.317) | 1.760(0.785,10.390) | <0.001 |

**Supplemental Table 2. Comparisons of demographics and clinical characteristics between the PJI and aseptic loosening patients in the train and validation cohort.**

| **Variable** | | **Train** | | |  | **Test** | | |
| --- | --- | --- | --- | --- | --- | --- | --- | --- |
|  |  | **Aseptic (N=202)** | **PJI (N=93)** | **P** |  | **Aseptic (N=92)** | **PJI (N=35)** | **P** |
| **Joint**  **(%)** | **Hip** | 100 (49.505%) | 43 (46.237%) | 0.602 |  | 56 (60.870%) | 16 (45.714%) | 0.125 |
|  | **Knee** | 102 (50.495%) | 50 (53.763%) |  |  | 36 (39.130%) | 19 (54.286%) |  |
| **sex**  **(%)** | **Male** | 90 (44.554%) | 40 (43.011%) | 0.804 |  | 45 (48.913%) | 15 (42.857%) | 0.541 |
|  | **Female** | 112 (55.446%) | 53 (56.989%) |  |  | 47 (51.087%) | 20 (57.143%) |  |
| **Age (years)** | | 66.000(59.000, 73.750) | 67.000(60.000, 75.000) | 0.708 |  | 65.000(58.750, 73.000) | 68.000(61.500, 75.000) | 0.356 |
| **Weight (Kg)** | | 60.000(55.000, 70.000) | 60.000(54.500, 68.000) | 0.568 |  | 60.000(54.000, 69.000) | 61.000(54.750, 67.500) | 0.517 |
| **BMI (Kg/m2)** | | 23.685(21.100, 25.955) | 23.440(21.300, 25.390) | 0.967 |  | 23.310(20.200, 25.495) | 24.240(20.670, 25.655) | 0.523 |
| **WBC (*109/L)** | | 5.885(4.942, 7.300) | 6.100(4.960, 8.550) | 0.173 |  | 5.558(4.500, 6.721) | 6.420(5.495, 8.630) | 0.005 |
| **HB (g/L)** | | 121.000(108.000, 133.000) | 117.000(106.000, 128.000) | 0.158 |  | 119.070(105.645, 130.250) | 120.180(107.500, 127.500) | 0.712 |
| **PLT (*109/L)** | | 143.000(120.000, 219.500) | 205.000(167.000, 256.000) | <0.001 |  | 180.965(123.000, 242.452) | 235.260(201.360, 276.525) | 0.017 |
| **ANG (*109/L)** | | 3.690(2.715, 4.620) | 3.980(2.840, 5.960) | 0.013 |  | 3.325(2.562, 4.470) | 3.870(3.545, 5.015) | 0.003 |
| **AL(*10^9^/L)** | | 1.670(1.270, 2.315) | 1.790(1.290, 2.970) | 0.231 |  | 1.850(1.330, 2.405) | 1.760(1.350, 3.380) | 0.392 |
| **N% (%)** | | 59.500(54.025, 67.125) | 65.400(59.100, 72.600) | <0.001 |  | 60.350(53.615, 66.650) | 65.700(60.140, 72.700) | <0.001 |
| **L %(%)** | | 25.950(18.700, 31.250) | 24.300(16.800, 31.000) | 0.449 |  | 26.465(19.355, 30.128) | 24.900(14.380, 27.300) | 0.041 |
| **DD (mg/ml)** | | 0.530(0.300, 0.970) | 1.770(1.270, 2.150) | <0.001 |  | 0.430(0.255, 0.762) | 1.560(0.840, 2.095) | <0.001 |
| **SE-IL-6 (ng/ml)** | | 4.370(4.060, 5.150) | 9.160(6.270, 12.730) | <0.001 |  | 4.360(4.060, 5.032) | 8.790(7.025, 11.875) | <0.001 |
| **SE-CRP (mg/ml)** | | 4.490(3.500, 6.415) | 30.500(14.600, 91.200) | <0.001 |  | 4.410(2.945, 7.075) | 22.200(10.185, 70.540) | <0.001 |
| **ESR (mm/h)** | | 27.500(15.000, 52.000) | 55.000(26.000, 85.000) | <0.001 |  | 27.500(12.785, 50.212) | 62.420(30.500, 93.605) | <0.001 |
| **CD64** | | 0.490(0.233, 0.808) | 2.250(1.360, 4.120) | <0.001 |  | 0.435(0.212, 0.831) | 2.510(1.120, 4.480) | <0.001 |
| **SF-CRP (mg/ml)** | | 5.035(2.887, 6.982) | 13.310(9.020, 20.460) | <0.001 |  | 4.529(2.763, 6.340) | 17.630(11.185, 25.745) | <0.001 |
| **IFN-γ (pg/ml)** | | 4.115(1.487, 16.665) | 5.350(1.890, 22.130) | 0.139 |  | 3.880(1.538, 16.535) | 12.270(3.465, 27.210) | 0.023 |
| **SF-IL-1β (pg/ml)** | | 32.670(15.262, 53.340) | 140.450(87.360, 213.690) | <0.001 |  | 29.282(11.131, 54.315) | 156.610(91.360, 207.845) | <0.001 |
| **SF-IL-2 (pg/ml)** | | 7.800(3.018, 13.568) | 7.020(2.900, 13.130) | 0.883 |  | 6.125(2.473, 13.915) | 5.710(1.745, 13.045) | 0.415 |
| **SF-IL-4 (pg/ml)** | | 2.350(1.603, 3.738) | 7.530(5.150, 14.770) | <0.001 |  | 2.034(1.425, 3.220) | 10.390(5.305, 15.080) | <0.001 |
| **SF-IL-6 (ng/ml)** | | 3.175(2.050, 6.917) | 14.280(7.550, 26.350) | <0.001 |  | 3.473(2.173, 8.489) | 11.260(8.140, 16.360) | <0.001 |
| **SFIL-8 (pg/ml)** | | 786.280(160.850, 1378.445) | 430.620(100.540, 1283.890) | 0.131 |  | 584.145(149.045, 1260.717) | 628.610(121.730, 1333.560) | 0.899 |
| **SF-IL-10 (pg/ml)** | | 2.285(1.255, 3.997) | 2.180(1.270, 4.950) | 0.861 |  | 2.165(1.048, 4.357) | 1.520(0.775, 4.555) | 0.455 |
| **SF-IL-12 (pg/ml)** | | 1.765(0.940, 4.103) | 1.770(0.760, 3.710) | 0.51 |  | 1.865(0.880, 3.702) | 1.420(0.970, 1.860) | 0.305 |
| **SF-IL-17 (pg/ml)** | | 1.500(0.780, 4.415) | 1.260(0.670, 3.810) | 0.348 |  | 1.275(0.725, 3.283) | 11.720(1.290, 17.860) | <0.001 |
| **PMN% (%)** | | 52.420(32.957, 59.580) | 72.290(52.400, 84.140) | <0.001 |  | 51.555(6.598, 64.308) | 76.610(70.155, 89.510) | <0.001 |

**Supplemental Table 3. Univariate and multivariate logistic regression analysis of the diagnosis of PJI in revision patients.**

| **Variables** | **Univariate logistic regression analysis** | | |  | **Multivariate logistic regression analysis** | | |
| --- | --- | --- | --- | --- | --- | --- | --- |
|  | **OR** | **95%CI** | **P** |  | **OR** | **95%CI** | **P** |
| **Joint** | 1.14 | 0.697-1.865 | 0.602 |  |  |  |  |
| **SE-IL-6** | 1.492 | 1.333-1.670 | ＜0.001 |  | 1.696 | 1.169-2.461 | 0.005 |
| **SE-CRP** | 1.118 | 1.083-1.153 | ＜0.001 |  | 1.039 | 0.930-1.161 | 0.496 |
| **ESR** | 1.021 | 1.012-1.029 | ＜0.001 |  | 1.035 | 0.990-1.082 | 0.132 |
| **CD64 index** | 3.785 | 2.628-5.451 | ＜0.001 |  | 2.186 | 1.169-4.090 | 0.014 |
| **SF-CRP** | 1.502 | 1.360-1.658 | ＜0.001 |  | 1.658 | 1.136-2.421 | 0.009 |
| **IFN-γ** | 1.008 | 0.996-1.020 | 0.199 |  |  |  |  |
| **SF-IL-1β** | 1.035 | 1.026-1.044 | ＜0.001 |  | 1.024 | 1.005-1.043 | 0.011 |
| **SF-IL-4** | 1.579 | 1.403-1.777 | ＜0.001 |  | 1.704 | 1.119-2.594 | 0.013 |
| **SF-IL-6** | 1.058 | 1.037-1.079 | ＜0.001 |  | 1.098 | 1.009-1.194 | 0.03 |
| **SF-IL-8** | 1 | 1.000-1.000 | 0.989 |  |  |  |  |
| **SF-IL-12** | 0.957 | 0.878-1.043 | 0.319 |  |  |  |  |
| **PMN%** | 1.024 | 1.013-1.034 | ＜0.001 |  | 1.06 | 1.004-1.120 | 0.036 |
| **DD** | 10.84 | 6.352-18.502 | ＜0.001 |  | 9.345 | 1.266-69.001 | 0.028 |

**Supplemental Table 4. Demographics and clinical characteristics of the External validation cohort.**

| **Variable** |  | **Overall (N=146)** | **PJI cohort (N=57)** | **Aseptic cohort (N=89)** | **P** |
| --- | --- | --- | --- | --- | --- |
| Gender (%) | Female | 82 (56.164%) | 32 (56.140%) | 50 (56.180%) | 0.996 |
|  | Male | 64 (43.836%) | 25 (43.860%) | 39 (43.820%) |  |
| Joint (%) | Hip | 76 (52.055%) | 31 (54.386%) | 45 (50.562%) | 0.652 |
|  | Knee | 70 (47.945%) | 26 (45.614%) | 44 (49.438%) |  |
| Age | | 66.500(60.000,74.000) | 68.000(62.000, 76.000) | 66.000(60.000, 73.000) | 0.420 |
| BMI | | 60.000(55.000,66.750) | 60.000(52.500, 66.000) | 60.000(55.000, 67.000) | 0.638 |
| Weight | | 23.440(21.027,25.810) | 23.050(20.770, 25.810) | 23.830(21.480, 25.810) | 0.470 |

**Supplemental Figure 1. Using LASSO logistic regression model to screen the potential predictors. (A)** The plot of partial likelihood deviance. **(B)** The plot of LASSO coefficient profiles. Each colorful curve represents the LASSO coefficient profile of a feature against the lnk sequence. The values above the figure represent the numbers of variables included in the model, given the corresponding k shown on the x-axis.

**
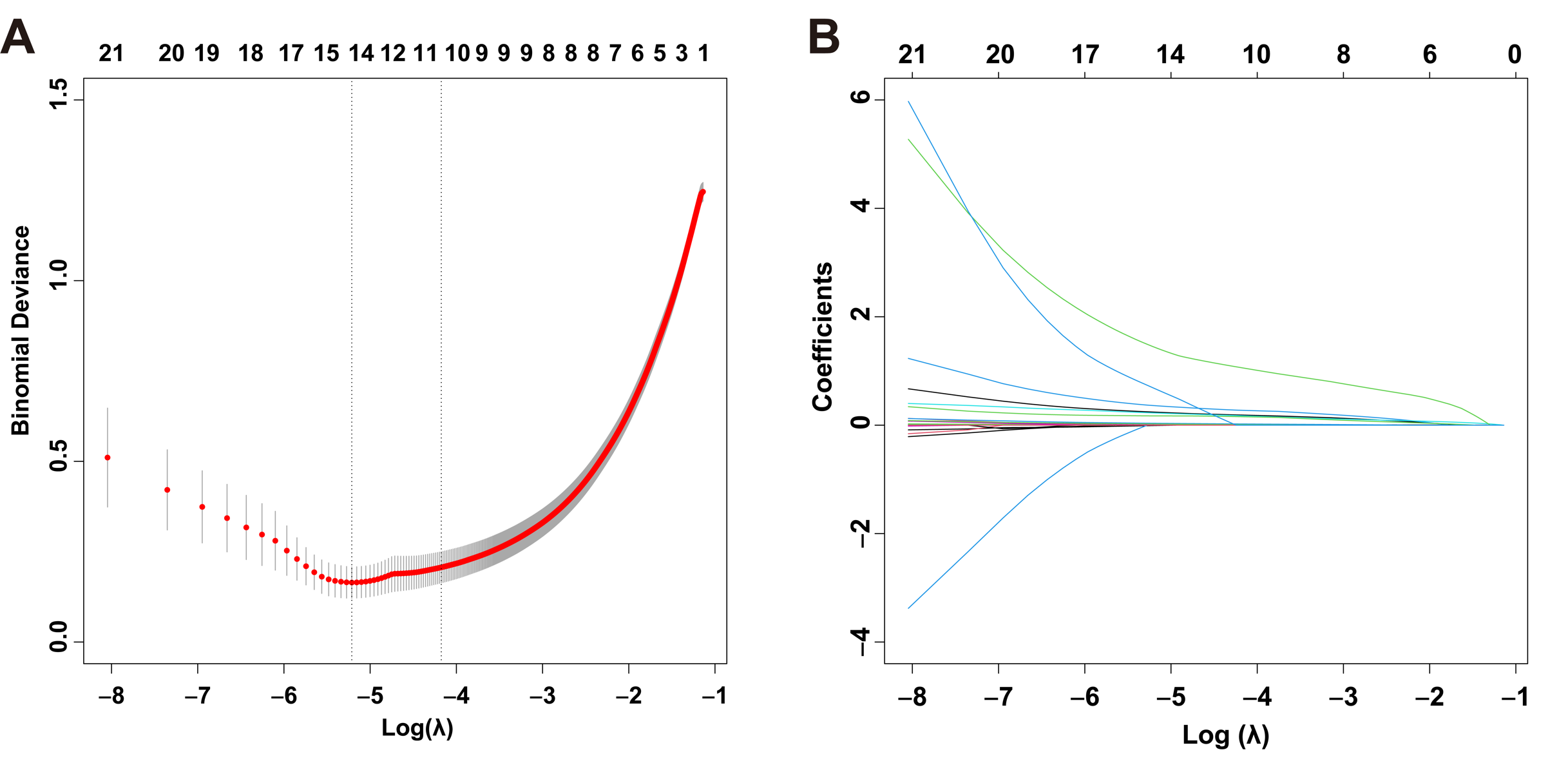
**
